# Prenatal diagnosis and perinatal outcomes of twin pregnancies disharmonious for one fetus with nuchal translucency above the 95th percentile

**DOI:** 10.1186/s13039-023-00659-9

**Published:** 2023-11-01

**Authors:** You Wang, Hang Zhou, Fang Fu, Ken Cheng, Ruibin Huang, Ru Li, Dongzhi Li, Can Liao

**Affiliations:** 1 The First Clinical Medical College,, Southern Medical University, Guangzhou, China; 2 Department of Prenatal Diagnostic Center, Guangzhou Women and Children’s Medical Center, Guangzhou Medical University, Guangzhou, China; 3 School of Medicine, South China University of Technology, Guangzhou, China

**Keywords:** Nuchal translucency, Twins, Prenatal diagnosis, MCT, DCT

## Abstract

**Objective:**

To assess prenatal diagnosis and pregnancy outcomes in twin pregnancies where one fetus has nuchal translucency (NT) above the 95th percentile.

**Method:**

In this retrospective analysis, 130 twin pregnancies (260 fetuses) in which one twin had an NT measurement above the 95th percentile while that of the other twin was normal were analyzed. Prenatal diagnostic results such as G bands, chromosomal microarray analysis, ultrasound findings, and pregnancy outcomes were reviewed.

**Results:**

Karyotype analysis and CMA results revealed that 15 (15.6 percent, 15/96) fetuses exhibited chromosomal abnormalities and that 13 fetuses were Variant of Uncertain Significance. Chromosome abnormalities were detected at a rate of 8.9% (5/56) in the DCT group and 25.0% (10/40) in the MCT group (*p* = 0.033, X2 = 4.571). 2 fetuses in DCT (3.9 percent, 2/51) and 4 fetuses in MCT (13.3 percent, 4/30) (*p* = 0.187) revealed structural abnormalities among the cases with normal prenatal diagnosis. Fetuses in the DCT group had an overall survival rate of 75.4 percent (95/126), whereas those in the MCT group had a survival rate of 60.4 percent (81/134) (*p* = 0.01, X2 = 6.636). According to the findings of Logistics regression analysis, NT thickening, maternal age and method of conception were all significant risk factors for chromosome abnormalities.

**Conclusion:**

In twin pregnancies with one fetus with NT above the 95th percentile, the prevalence of fetal structural abnormalities of the MCT group and the DCT group were comparable. Pregnant women’s age and mode of pregnancy are risk factors for chromosomal abnormalities.

## Introduction

Pregnant women’s age and mode of pregnancy are risk factors for chromosomal abnormalities. The nuchal translucency (NT), maternal age, mode of pregnancy, and chorionicity are the risk factors for fetal survival [1–3]. Notably, NT is a biomarker of early fetal pregnancy that is relatively specific and associated with common aneuploidy, microdeletion, microrepetition syndrome, and poor prognosis. The thickness of the liquid buildup in the subcutaneous tissue of the fetus's posterior neck is referred to as the "fetal neck transparent layer" [4]. The chromosome is depicted on ultrasound imaging as an echoless spot in the subcutaneous tissue of the posterior neck of the fetus. Recently, it has drawn much attention from medical professionals recently. In some circumstances, the translucent layer around the neck that is translucent may thicken [4]. Chromosomal abnormality markers and increased NT thickness have been linked to widespread structural defects, genetic syndromes, high abortion risk, and fetal intrauterine death [5]. The risk increases in direct proportion to the thickness of the transparent covering around the neck. Chromosomal abnormality markers and increased NT thickness are linked to widespread structural defects, genetic syndromes, high abortion risk, and fetal intrauterine death [6]. This risk increases in direct proportion to the thickness of the transparent covering around the neck [7].

The majority of monocytic studies have concluded that a variety of genetic variables, ranging from mutations in a single nucleotide to genetic issues affecting millions of base pairs and structural defects, are responsible for NT thickness [5, 7, 8]. However, only a very small number of studies have reported on twins where one fetus with nuchal translucency is above the 95th percentile. Furthermore, many pertinent studies have concentrated on the relationship between NT thickness in monochorionic twins and other twin pregnancies problems such as twin-to-twin transfusion syndrome (TTTS) [9, 10]. Therefore, the prenatal outcomes and perinatal outcomes for a twin with NT > 95th percentile have not been well characterized.

Our study aimed to investigate the prenatal diagnosis and pregnancy outcomes of twin pregnancies with a fetal NT measurement value > 95th percentile in dichorionic twins (DCT) and monochorionic twins (MCT).

## Materials and methods

### Research participants and management

The study was conducted in accordance with the ethical guidelines of the Ethics Committee of the Guangzhou Women and Children's Medical Center in Guangdong province. From September 2017 to March 2022, a total of 996 twin pregnancies (771 DCTs, 225 MCTs) came to our prenatal diagnosis center for prenatal ultrasound scans. NT thickening was identified by prenatal ultrasound in 175 cases (97 cases of DCTs, 78 cases of MCTs). The inclusion criteria for this study were: (I) twin live pregnancies; (II) One fetus with an NT above the 95th percentile (NT range: 3.0–6.0 mm); (III) Able to successfully follow up. A total of 45 cases were excluded from this study as they did not meet the inclusion criteria. The specific reasons for exclusion were as follows: 13 cases of one of the twins arrested developing, 8 cases of fetal cystic hygroma, 5 cases of NT thickening in both fetuses, and 19 cases of lost follow-up.

A total of 130 cases (260 fetuses) met the inclusion criteria for this study, including 67 MCTs (134 fetuses) and 63 DCTs (126 fetuses), each of which had one fetus with an NT above the 95th percentile. Among the 67 MCT, 28 cases (56 fetuses) successfully underwent invasive prenatal diagnosis, 37 cases of parents refused prenatal diagnosis, and 2 cases failed the test due to poor DNA quality. Among the 63 DCTs, 20 cases (40 fetuses) successfully underwent invasive prenatal diagnosis, 40 cases of parents refused prenatal diagnosis, and 3 cases failed the test due to insufficient/poor DNA quantity. Overall, invasive prenatal diagnosis was performed in 96 fetuses in 48 cases.

The two-dimensional probe frequency ranges from 2.5 to 5.0 MHz and the three-dimensional volume probe frequency ranges from 4.0 to 8.0 MHz on the GE Volusion E10 color Doppler ultrasound diagnostic device. Our fetal anatomy evaluation scan program complies with the 2013 early pregnancy scan recommendations of the International Society of Obstetrics and Gynecology Ultrasound (ISUOG). When ultrasound was used to detect fetal structural abnormalities, we excluded parabiotic twins and one of the twins without a heart. As it is difficult to accurately measure NT and crown-rump length (CRL) when these two complications are combined, they were excluded [9]. We combined cytogenetic results, chromosomal microarray analysis (CMA), and ultrasound results to retrospectively analyze fetal pregnancy outcomes. Ultrasonography in the early stages of pregnancy and placenta inspection after birth was used to identify chorionic properties. Placental fusion at the junction of the two gestational sacs was defined as the “entering” sign (twin-peaks or “lambda” sign) of dichorionic twins and a thin, T-shaped septum between monochorionic chorionic twins with only one placenta. Placental data of pregnant women after delivery were examined if available. In DCT, two placentas can be seen within each chorion or fused into one placenta where the boundary is clear and there is no blood vessel traffic. The diaphragm between the twins includes two layers of chorion and two layers of amniotic membrane. There was only one placenta in monochorionic twins, with vascular anastomosis between the two fetuses on the surface and interior of the placenta, and the diaphragm between the two fetuses had two layers of the amniotic membrane without chorion. Women with monochorionic, diamniotic (MCDA) twin pregnancies and suspected TTTS or selective intrauterine growth restriction (sIUGR) may consider laser coagulation, laser ablation, or selective fetal reduction of placental vascular anastomosis under fetal endoscopy. sIUGR was defined as the estimated body weight of two fetuses having ≥ 25% difference, and the smallest difference was less than the 5th percentile.

### Cytomolecular genetic analyses

CMA was performed using the CytoScan 750 K Array (Affymetrix Inc., Santa Clara, CA, USA), which contains 750,436 25-85mer oligonucleotide probes, including 200,436 single-nucleotide polymorphic (SNP) probes and 550,000 non-polymorphic (NP) probes (0.1 Mb resolution). The copy number variation (CNV) threshold was set to 100 kb, and the number of markers was ≥ 50 kb. According to the ACMG CNV guidelines (specific guidelines), CNVs identified by CMA were divided into pathogenic CNVs, variants with uncertain significance (VOUS), and benign CNVs. CMA was used to evaluate the DNA of both parents to confirm whether the pathogenic CNVs and VOUS were new or inherited. Chromosomal karyotype analysis experiment was performed to select the cells at the vigorous proliferation stage for colchicine treatment so that the cell division was stopped at the middle stage, to obtain enough mitotic cells. After low permeability, fixation, preparation, and staining, the cells were observed under a microscope. Chromosomal banding karyotype analysis is performed on the chromosome specimen obtained by banding technology so that the chromosomes along the longitudinal axis show a certain number of different degrees of coloring, and various widths of light and dark bands. Each chromosome has a unique and constant stripe, depending mainly on the interaction between DNA, proteins, and dyes. Different banding techniques produced different bands on the chromosomes. The G-banding technique involves digestion of chromosome specimens with trypsin and staining with Giemsa.

### Pregnancy outcomes

Details concerning the maternal demographics, follow-up prenatal process, and pregnancy outcomes were obtained from the existing medical record system of the medical center. Alternatively, the outcome data of all patients could be routinely obtained from the delivery unit, the patient, or the referral doctor.

### Statistical analysis

Statistical software packages SPSS24.0 (IBM) and Minitab19.0 were used for data analysis. All qualitative data were expressed as n (%). All continuous data were retested for normality, and non-normally distributed data were tested using a non-parametric test. The Chi-square test was used to evaluate the qualitative data. We used logistic regression analysis to evaluate the potential independent variables for detecting fetal survival rate and chromosome abnormality rate. *P* < 0.05 (two-tailed test) was considered statistically significant.

## Results

### Study population

This study covered 130 cases of NT thickening (NT range: 3.0–6.0 mm) twin pregnancies (260 fetuses), including 63 cases of DCTs (126 fetuses) and 67 cases of MCTs (134 fetuses) from 1 January 2015 to 1 January 2022 in our center. The demographic characteristics and results of the twin pregnancies are shown in Table 1.

**Table 1:** Patients’ characteristics

Variables	DCDA	MCDA	MCMA
Fetus	126	122	12
MA	31.7 ± 5.3	29.1 ± 4.4	31 ± 7.1
GA	12.74 ± 0.4	12.9 ± 0.6	12.8 ± 0.6
IVF	56(44.4%)	4(3.3%)	0
OI	8(6.3%)	0	0
SP	51(40.5%)	112(91.8%)	12(100.0%)
Villocentesis	12(9.5%)	0	0
Amniocentesis	44(34.9%)	38(31.1%)	2(16.7%)
CMA	56(44.4%)	38(31.1%)	2(16.7%)
Survival	95(75.4%)	78(63.9%)	3(25.0%)
Structural abnormalities	15(11.9%)	11(9.0%)	4(33.3%)
Chromosomal abnormality	6(10.7%)	10(25.0%)	0

*SP* Spontaneous. *MA* Mean maternal age, *GA* Gestational age, *OI* Ovulation induction, *IVF* In-vitro fertilization

*CMA* Chromosomal microarray analysis

### Prenatal diagnosis results

Table 2 shows the clinical and chromosomal characteristics. Chromosomal karyotype analysis and CMA were performed simultaneously for one fetus with NT thickening in each pair of twins. The two detection results for chromosomal aneuploidy and chromosome large segment abnormalities were comparable. In addition, the abnormal detection rate of CMA was 8.1% higher than that of karyotype analysis. The karyotype analysis and CMA showed significant chromosomal abnormalities in 15 fetuses, including 9 cases of trisomy 21, 3 cases of Turner syndrome, 4 cases of pathological CNVs, and 2 cases of mosaic (1 case mos 45, X^2^/46, XY [98] level III, and 1 case mos 46, X, + mar [74]/45, X [26] level III) (see Table 2). Table 3 shows 13 cases of clinical characteristics with VOUS. In general, the incidence of chromosomal abnormalities in the DCT group was 8.9% (5/56), and that in the MCT group was 25.0% (10/40) (*P* = 0.033). This difference was statistically significant.

**Table 2:** Clinical characteristics with chromosomal abnormalities in one fetus with NT above the 95th percentile

NO	MA	GA	CM	NT	NASAL BONE	Twins	Karyotype	Interpretation of CMA Results	Outcomes
1	36	13.3	SP	8.2	0	MCDA	45, X	The deletion of 155.07 Mb was detected in chromosome Xp22.33q28, pathogenic	Induced labor
2	36	13.3	SP	1.4	0	MCDA	46, X, + mar	2.20 Mb microdeletion was detected in chromosome Xp22.33, pathogenic	Induced labor
3	27	12.4	SP	3.11	1	MCDA	47, XN, + 21	Chromosome 21q11.2q22.3 detected 33.06 Mb repeats, 21 trisomy syndrome, pathogenic	Induced labor
4	27	12.4	SP	2.95	0	MCDA	47, XN, + 21	Chromosome 21q11.2q22.3 detected 33.06 Mb repeats, 21 trisomy syndrome, pathogenic	Induced labor
5	28	12.5	SP	3.14	1	MCDA	47, XN, + 21	Chromosome 21q11.2q22.3 detected 33.06 Mb repeats, 21 trisomy syndrome, pathogenic	Induced labor
6	28	12.5	SP	2.89	0	MCDA	47, XN, + 21	Chromosome 21q11.2q22.3 detected 33.07 Mb repeats, 21 trisomy syndrome, pathogenic	Induced labor
7	29	12.4	SP	4.12	0	MCDA	47, XN, + 21	Chromosome 21q11.2q22.3 detected 33.06 Mb repeats, 21 trisomy syndrome, pathogenic	Induced labor
8	29	12.4	SP	3.96	1	MCDA	47, XN, + 21	Chromosome 21q11.2q22.3 detected 33.06 Mb repeats, 21 trisomy syndrome, pathogenic	Induced labor
9	32	13.3	SP	8.2	0	DCDA	45, X	The deletion of 155.07 Mb was detected in chromosome Xp22.33q28, pathogenic	Induced labor
10	32	13.3	SP	1.7	0	MCDA	47, XN, + 21	Chromosome 21q11.2q22.3 detected 33.08 Mb repeats, 21 trisomy syndrome, pathogenic	Induced labor
11	40	12.4	SP	5.12	1	DCDA	46, XY	8.18 Mb deletion was detected in chromosome Y q11.223q11.23, pathogenic	Induced labor
12	40	12.4	SP	3.65	1	DCDA	47, XN, + 21	Chromosome 21q11.2q22.3 detected 33.06 Mb repeats, 21 trisomy syndrome, pathogenic	Induced labor
13	40	12.9	SP	3.09	1	DCDA	47, XN, + 21	Chromosome 21q11.2q22.3 detected 33.06 Mb repeats, 21 trisomy syndrome, pathogenic	Induced labor
14	40	12.9	SP	3.76	0	DCDA	47, XN, + 21	Chromosome 21q11.2q22.3 detected 33.06 Mb repeats, 21 trisomy syndrome, pathogenic	Induced labor
15	32	13.3	SP	3.2	0	DCDA	46, X, + mar	Microdeletion of 221 kb was detected in chromosome Xp22.33, pathogenic	Survival

*SP* Spontaneous. *MA* Mean maternal age, *GA* Gestational age, *CM* Conceived method, *CMA* Chromosomal microarray analysis

**Table 3:** Clinical characteristics with VOUS in one fetus with NT above the 95th percentile

NO	MA	GA	CM	NT	NASAL BONE	Twins	Karyotype	Interpretation of CMA Results	Outcomes
1	29	13.3	SP	2.25	0	DCDA	46, XN	256 kb microdeletion was detected in chromosome 1p21.1	Ceaserian section
2	35	13.1	IVF	2.46	0	DCDA	46, XN	297 kb microduplication was detected in chromosome 18q22.1	Ceaserian section
3	32	13.1	IVF	2.21	0	DCDA	46, XN	391 kb microduplication was detected in chromosome 3q26.32	Normal labour
4	31	13.4	SP	8.01	0	DCDA	46, XN	614 kb microduplication was detected in chromosome 3p13p12.3	Premature delivery
5	31	13.4	SP	2.7	0	DCDA	46, XN	618 kb microduplication was detected in chromosome 3p13p12.3	Premature delivery
6	40	13.7	SP	7.5	1	DCDA	46, XN	500 kb microduplication was detected in chromosome 5p14.2	Normal labour
7	31	13.9	IVF	2.7	0	DCDA	46, XN	118 kb microdeletion was detected in chromosome 2q25	Ceaserian section
8	38	13.9	IVF	3.2	0	DCDA	46, XN	368 kb microduplication was detected in chromosome 7p22.2	Ceaserian section
9	38	13.8	IVF	1.6	0	DCDA	46, XN	368 kb microduplication was detected in chromosome 7p22.2	Ceaserian section
10	36	12.7	SP	2.77	0	DCDA	46, XN	1.37 Mb microduplication was detected in chromosome 15q13.2q13.3	Normal labour
11	34	13.1	IVF	3.11	0	DCDA	46, XN	300 kb microduplication was detected in chromosome 18q22.1	Normal labour
12	34	13.1	IVF	2.46	0	DCDA	46, XN	300 kb microduplication was detected in chromosome 18q22.1	Normal labour
13	32	12.9	SP	1.48	0	MCDA	46, XN	256 kb microdeletion was detected in chromosome 3p14.2	Ceaserian section

*SP* Spontaneous. *MA* Mean maternal age. *GA* Gestational age, *CM* Conceived method, *CMA* Chromosomal microarray analysis

0 Nasal bone exists; 1 Nasal bone loss

### Perinatal outcomes

**Fig. 1:**
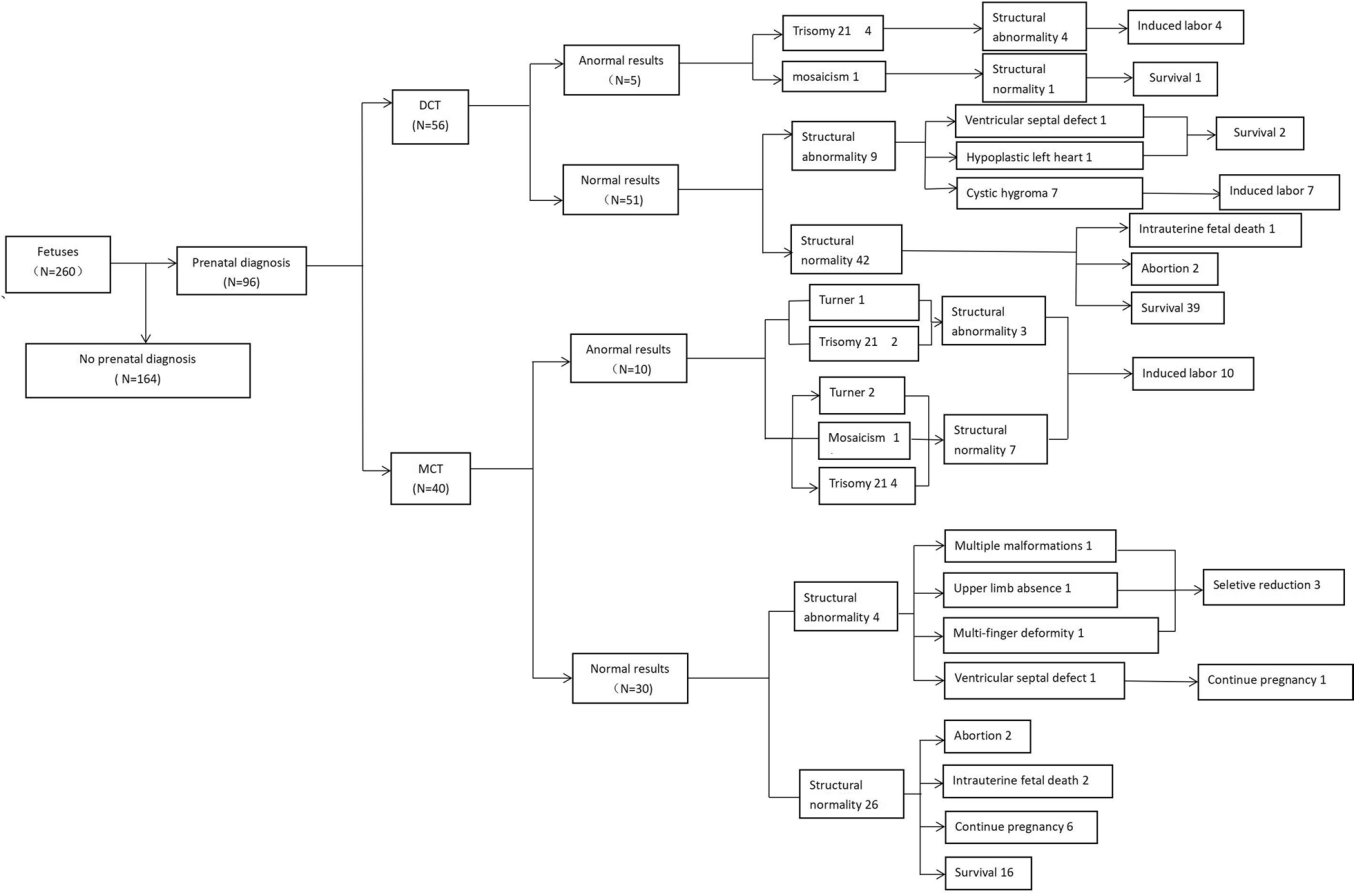
Flow chart of this study

**Fig. 2:**
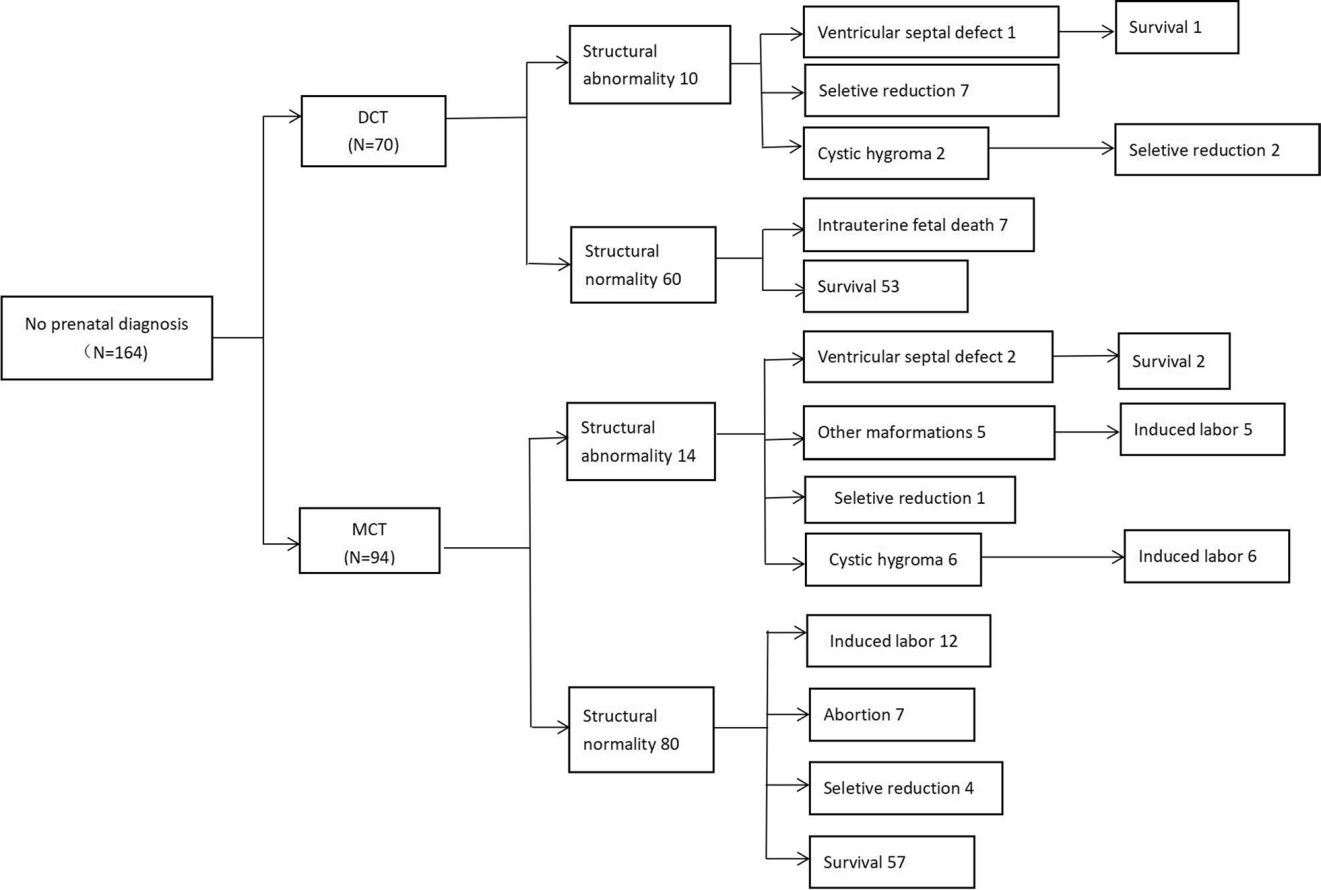
Supplementary drawing of Fig. 1

In this study, there were 31 cases of structural abnormalities, including cardiac malformations, cleft lip, and palate et al. Structural abnormalities were found in 22 of all DCT pregnancies (17.4%, 22/126) and 21 of all MCT pregnancies (15.6%, 21/134), *p* = 0.698 (see Fig. 2). Among twin pregnancies in which one fetus had an NT > 95th and a normal prenatal diagnosis: in the DCT group, the fetal survival rate was 80.4% (41/51); In the MCT group, the overall fetal survival rate was 76.7% (23/30) (see Fig. 1). However, the overall survival rate of all DCT fetuses was 75.4% (95 / 126), and the overall survival rate of MCT fetuses was 60.4% (81/134) (*P* = 0.01, X2 = 6.636), with indicated significant differences between the two groups. Detailed information on prenatal outcomes and follow-up is documented in the flowchart in Fig. 1. The supplementary information is recorded in the flowchart of Fig. 2.

### Statistical analysis

Table 4 shows the logistics regression analysis in risk factors of different classifications. In the cases of prenatal diagnosis, Logistic regression analysis showed that NT value, maternal age, and pregnancy mode were the high-risk factors for fetal chromosomal abnormalities. Notably, NT was a high-risk factor for fetal structural abnormalities, and NT value, maternal age, chorionic property, and pregnancy mode were high-risk factors for adverse pregnancy outcomes. NT was a high-risk factor for chromosomal abnormalities, the odds ratio (OR) of which was statistically significant (*P* < 0.05) (Table 4). Additionally, the incidence of fetal chromosomal abnormalities and adverse pregnancy outcomes was significantly different between the MCT and DCT groups (Table 5).

**Table 4:** Comparison of chromosomal results and clinical outcomes between DCT and MCT group in one fetus with NT above the 95th percentile

	Chromosome abnormality rate	Structure abnormality rate	Adverse pregnancy outcomes rate
DCT	8.9% (5/56)	17.5% (22/126)	24.6% (31/126)
MCT	25% (10/40)	15.7% (21/134)	39.6% (53/134)
*X*^2^ value	4.571	1.51	6.636
*P* value	0.033	0.698	0.010

**Table 5:** Logistics regression analysis in risk factors of different classifications in one fetus with NT above the 95th percentile

Classification	NT		MA		Chorionic property		Conceived methods	
	OR (95%CI)	*P*	OR (95%CI)	*P*	OR (95%CI)	*P*	OR (95%CI)	*P*
Chromosome abnormality	0.822	0.192	1.212	0.005	0.665	0.588	0.175	0.020
	(0.612–1.104)		(1.058–1.387)		(0.152–2.907)		(0.040–0.760)	
Structural abnormality	1.251	0.002	0.984	0.750	0.712	0.572	0.382	0.155
	(1.089–1.437)		(0.888–1.089)		(0.219–2.314)		(0.101–1.440)	
Adverse pregnancy outcome	1.875	0.001	0.920	0.016	1.171	0.025	6.463	0.000
	(1.319–2.428)		(0.860–0.985)		(1.018–1.342)		(2.576–16.217)	

## Discussion

Unlike most previous studies that focused on the association between discordance of increased NT and TTTs in MCT, this study focused on prenatal diagnosis and perinatal outcomes in twin pregnancies with one twin at the NT > 95th percentile in both MCT and DCT. In this study, twin pregnancies where one fetal NT thickening was observed were included. The results of chromosome karyotype analysis and CMA examination showed that the risk of chromosomal abnormalities in the twins with one fetal NT thickening was as high as 15.63% (15/96). Fetuses in the DCT group had an overall survival rate of 75.4% (95/126), whereas those in the MCT group had a survival rate of 60.4% (81/134) (*P* = 0.01, X2 = 6.636). According to the findings of the logistics regression analysis, NT thickness, maternal age, and pregnancy mode were all significant risk factors for chromosomal abnormalities. The NT value, age of the pregnant women, zygosity, and mode of pregnancy were risk factors for unfavorable pregnancy outcomes. NT is a risk factor for fetal structural abnormalities. Overall, these results could provide support and insight to aid in genetic counseling and prognosis in prenatal twins where one fetus has thickened NT.

As described above, this study found that NT thickness was the main high-risk factor affecting the rate of chromosomal abnormalities (OR = 1.891, 95% CI 0.612–5.099). Discordance in NT thickness is commonly related to twin pregnancies. This would be different with increased NT indication in singleton. The level of NT thickness would be related to a different risk of aneuploidy. Many studies have confirmed that NT thickening is associated with fetal aneuploidy chromosome karyotype abnormalities in singleton, with trisomy 21 being the most common. Trisomy 21 was found in both the DCT and MCT groups in our study, which is inconsistent with the results of previous studies [9]. Two cases of MCT had discordant karyotype results, including one for Turner syndrome and one for mosaic. This may be related to several mechanisms. Heterokaryotic monozygotic twins are an uncommon occurrence associated with mitosis after zygote formation (no division or late cell division lag). First, although the zygote may initially have a normal karyotype (46 chromosomes), due to a delay in mitosis, late cell division results in triploid (47 chromosomes) and mono-chromosome cells (45 chromosomes) as well as triploid cells. On the other hand, the zygote may be triploid initially (47 chromosomes), but due to the division of mitosis, a lagging diploid (46 chromosomes) may form at the final step of cell division; this process is known as triploid rescue (4). Three to five primordial cells typically serve as the embryonic source during the blastocyst stage. Monochorionic twins result from division at this stage. The separation of aneuploid progenitor cells and the proliferation of dominant diploids may result from any of these cells undergoing incorrect mitosis. This aneuploid twin will not leave any detectable residual if it is not viable and will be absorbed. However, MCT may develop from this aneuploid.

NT thickening is associated with an increased risk of chromosomal abnormalities. Our data showed a higher incidence of chromosomal abnormalities in pregnancies with NT thickening in one of the twins (15.63%, 15/96), particularly in the MCT group (25.0%, 10/40). With an increase in the NT value, the incidence of fetal chromosomal abnormalities also increased. Logistic regression analysis showed that the risk of chromosomal abnormalities increased 1.891 times for each 1 mm increase in NT value in the twin NT thickening, consistent with the reports in the literature at home and abroad. It is worth noting that because Karyotype analysis and CMA results were not available in every case, the incidence of abnormal karyotype analysis and CMA results could be severely influenced by selection bias, we cannot suppose those cases without karyotype analysis and CMA results were normal.

In our study, early pregnancy NT is one of the most important predictors of adverse fetal outcomes, and NT values correlate with maternal age at term and increase with increasing fetal CRL. It has been suggested that pregnancies in which one of the twins has NT thickening are closely associated with fetal chromosomal abnormalities. The results of this study are consistent with those of previous studies [1, 8, 9]. The detection rate of pathogenic CNVs in fetuses with thickened NT and normal karyotype was 2.7% to 12.8% in the combined literature. The reason why the results of this study are not fully consistent with other related literature may be attributed to the differences in case inclusion criteria, fetal NT value distribution, and the CMA technology platforms used in different studies [7, 8].

Additionally, logistic regression analysis revealed that the risk of fetal structural abnormalities increased with fetal NT thickening in twin pregnancies with NT thickening in one fetus. Ultrasound screening in early pregnancy has a positive effect on reducing birth defects as it can detect certain serious structural abnormalities of the fetus, such as total forebrain, umbilical bulge, abdominal cleft, single ventricle, and severe left heart developmental abnormalities. Special attention should be given to the fact that cardiac anomalies are the most common structural anomalies in twins with one fetal NT thickening. Some studies have shown that the incidence of cardiac structural anomalies is positively correlated with NT thickness[5]. Heart failure may be a contributing factor in this mechanism. Venous reflux problem causes an increase in jugular venous pressure during heart failure. When the jugular vein's internal pressure is greater than that of the lymphatic vessel, the return of lymphatic fluid to the jugular vein is prevented, and an excessive buildup of lymphatic fluid in the neck results in the formation of a thickening of the cervical transparent layer [11]. Our study suggests that if fetal NT thickening is found in twin pregnancies, attention should be paid to early pregnancy ultrasound scans to rule out serious structural cardiac anomalies including single ventricle and severe left heart developmental anomalies. In addition, fetal echocardiography mid-pregnancy should be performed to screen for associated cardiac and macrovascular anomalies.

CNVs of unclear significance are challenging for clinicians to manage, so physicians must properly consult with the patient before and after prenatal diagnosis. Compared with karyotype analysis, CMA can detect additional microdeletions or microduplications of chromosome structures and genes involved in the mutation section, location, and fragment size. Our results showed that the overall survival rate of pregnant women with DCT was higher than that of pregnant women with MCT. This differs from the results of previous studies [1]. This is primarily due to the following reasons: in MCT, there is a higher risk of chromosomal abnormalities, unpredictable vascular traffic anastomosis between the twins, and uneven distribution of individual placentas leading to increased complications specific to twin pregnancies including TTTS or sIUGR. We found that the incidence of chromosomal abnormalities was lower in the DCT group (10.7%) than in the MCT group (25.0%) in twin pregnancies in which one fetus has an NT value of > 95th percentile. The high risk of chromosome abnormality in the MCT group may be brought on by zygotes in a state of chromosome mosaicism before zygote division, gene mutation following zygote division, a deviation in the X chromosome's inactivation pattern, a change in the genomic imprinting pattern, changes in genes' methylation patterns, or histone modifications ^(12, 13)^. This differs from previous studies [1]. The possible reason for the difference between the results of these two studies is the slightly larger sample size of the present study (260 vs. 93).

In addition, due to the hemodynamic imbalance caused by the vascular anastomoses between the two sides of the placenta, MCTs are more likely to experience perinatal death and morbidity than DCTs [11]. Moreover, this study an incidence of sIUGR of 13% in MCTs in late pregnancies. When predicting the incidence of TTTS in MCTs, NT thickening in one twin or a difference in NT thickness between two twins of less than 0.6 mm had a certain sensitivity (50%) and a high specificity (92%) [8, 14]. Future TTTS is more than 90% more likely if fetal NT thickening is accompanied by venous catheter (DV) reflux.

Logistic regression analysis yielded a lower survival rate in the in vitro fertilized (IVF) group than in the naturally conceived group (*P* = 0.000). Based on these results, the awareness of the increased obstetric risks of IVF may mean that these pregnancies should be managed as "high risk" [6, 9]. Data obtained through a rigorous systematic evaluation in 2004 showed that IVF-conceived pregnancies have an increased risk of preterm birth, very preterm birth, low birth weight, very low birth weight, and small for gestational age (SGA) compared to those following natural conception, which thus affects fetal survival [13].

In the DCT group, NT thickening may be due to a potential mechanism such as monocytosis. Indeed, around 10 to 13 + 6 weeks into a typical embryo's development, the cervical lymphatic veins and jugular sinus were joined. A small amount of lymphoid tissue was collected from the neck before the cervical lymphatic and jugular sinuses were joined. A transient reflux condition was observed, resulting in brief thickening of the transparent layer of the neck. After 14 weeks, a typical fetus is no longer viable [12, 13] If the neck lymph duct and neck sinus venous connection take longer than expected, it will cause considerable neck lymph reflux issues [5], excessive lymphatic fluid buildup, and thickening of the transparent layer of the neck. In contrast, in the MCT group, NT thickening was not only associated with chromosomal abnormalities but may also be due to complications, such as TTTS or sIUGR, resulting from shared placental circulation leading to fetal loss, which was also observed in our study.

Unlike most previous studies [1, 5, 7, 15] that focused on the association between increased NT and TTTS in MCT inconsistency, the present study focused on prenatal diagnosis and pregnancy outcomes in twin pregnancies in which one of the twin fetuses had an NT > 95th percentile. Several previous studies [1, 5, 7, 15] have shown that NT-thickened fetuses are also associated with a slightly increased risk of structural malformations, miscarriage, and other adverse pregnancy outcomes. Our study showed a significant difference in the incidence of adverse pregnancy outcomes between MCTs and DCTs, with adverse pregnancy outcomes being more common in MCT patients. Shi et al. [1] did not describe the view that fetal conception is a risk factor affecting the overall survival rate of the fetus.

Some limitations of this study must be examined. First, this was a retrospective single-center cohort, with some inherent risk of bias and a small sample size. Second, in the case of early fetal loss, no pathological examination of the fetus was performed, so the cause of fetal death or whether the fetus was abnormal was unknown. Furthermore, monogenic genetic disorders including Noonan syndrome have been previously reported to be associated with fetal NT thickening in early pregnancies, but this study did not use a WES line to further validate fetuses with normal karyotype and CMA results. Therefore, data related to monogenic NT thickening disorders in one of the associated twin fetuses are lacking, and some monogenic genetic disorders may have been missed. Finally, it is a retrospective study- that may have recall bias, and it is difficult to confirm IVF contributed to nearly 50% of DCDA twins or PGT-A contributed to less commonly aneuploidy or genetic defects. Additional multicenter prospective studies are necessary to assess the prenatal and perinatal outcomes of twin pregnancies with abnormal NT.

In summary, we have demonstrated in twin pregnancies that when one twin's NT is above the 95th percentile, the incidence of chromosomal abnormalities is lower in the DCT group than in the MCT group. Furthermore, the overall survival rate of the DCT group was higher than that of the MCT group, while the risks of fetal structural malformations were comparable. NT thickening and zygosity are high-risk factors for chromosomal abnormalities and adverse pregnancy outcomes. The low survival rate of fetuses with NT thickening in the MCT group was mainly due to the high incidence of chromosomal abnormalities, fetal structural abnormalities, and unique complications such as sIUGR.

## Data Availability

The original contributions presented in the study are included in the article, further inquiries can be directed to the corresponding author.
